# Exploring poly-environmental loads in psychosis: the applications of exposome score for schizophrenia and exposome wide association studies

**DOI:** 10.1192/j.eurpsy.2026.10323

**Published:** 2026-07-31

**Authors:** A. Arias-Magnasco

**Affiliations:** Department of Psychiatry and Neuropsychology, Maastricht University, Maastricht, Netherlands

## Abstract

**Abstract:**

Environmental risk factors play a central role in the development of psychosis; however, psychiatric research has traditionally examined these exposures in isolation. This has limited our ability to capture the complex, cumulative, and intercorrelated nature of environmental experiences across the life course.

In this talk, we will outline the shift from single-exposure models toward a poly-environmental perspective grounded in the exposome concept. We will first review how environmental liability has historically been investigated in psychiatry, and then introduce contemporary strategies to quantify environmental risk, ranging from simple cumulative indices to scores weighted by meta-analytic effect sizes. Particular emphasis will be placed on the Exposome Score for Schizophrenia (ES-SCZ), which integrates multiple environmental exposures while explicitly accounting for their intercorrelations. We will describe how the ES-SCZ is constructed, what distinguishes it from previous environmental risk scores, and why modeling exposure dependency is critical for understanding psychosis risk.

We will then review recent empirical findings applying the ES-SCZ across the psychosis spectrum, including subclinical psychotic experiences and clinical outcomes. Finally, we will discuss agnostic, data-driven approaches such as exposome-wide association studies (ExWAS) as complementary tools to hypothesis-driven research.

**Disclosure of Interest:**

None Declared

